# Proposed Environmental Risk Management Elements in a Carpathian Valley Basin, within the Roşia Montană European Historical Mining Area

**DOI:** 10.3390/ijerph18094565

**Published:** 2021-04-25

**Authors:** Doru Bănăduc, Angela Curtean-Bănăduc, Kevin Cianfaglione, John Robert Akeroyd, Lucian-Ionel Cioca

**Affiliations:** 1 Applied Ecology Research Center, Lucian Blaga University of Sibiu, I. Raţiu Street 5-7, 550012 Sibiu, Romania; angela.banaduc@ulbsibiu.ro; 2 UMR UL/AgroParisTech/INRAE 1434 Silva, Faculté des Sciences et Techniques, Université de Lorraine, BP 70239, 54506 Vandoeuvre-lés-Nancy, France; kevin.cianfaglione@gmail.com; 3 Sherkin Island Marine Station, Sherkin Island, Skibbereen, Ireland; john@johnakeroyd.co.uk; 4 Department of Industrial Engineering and Management, Faculty of Engineering, Lucian Blaga University of Sibiu, Bd. Victoriei No. 10, 550024 Sibiu, Romania; 5 Academy of Romanian Scientists, 3 Ilfov Street, Sector 5, 010071 Bucharest, Romania

**Keywords:** human impact, lentic and lotic ecosystems, aquatic macroinvertebrates, fish, threats, risk management, Corna Basin

## Abstract

Non-ferrous metals mining activities have long accompanied people, and began in the study area of South East Europe over 2000 years ago. The environment quality is significantly affected by both historic mining activities and contemporary impacts. All these problems, inducing synergic negative effects on local organism communities, have created a chronic state of pollution. The Corna Valley has one of the oldest historical human impacts in Romania due to the influence of mining. Fish and benthic macroinvertebrates have exhibited significant responses to long term mining effects on lotic systems. The analysis of macroinvertebrate communities, correlated with the lack of fish and some biotope characteristics, indicates that the Corna River presents a variety of categories of ecological status between sectors. The lack of fish reveals the poor ecological conditions. Technical and management solutions are proposed here to diminish the historical environmental problems and to avoid future ecological accidents, especially in an attempt to improve any construction plan concerning a possible new de-cyanidation dam and lake. Fish and benthic macroinvertebrates have exhibited significant responses to long term mining effects on lotic systems. Two management zones were identified, an upper zone which can be used as a reference area and a lower zone, where pollution remedial activities are proposed.

## 1. Introduction

Mining activities have accompanied people from prehistory, from the Neolithic age (e.g., Grimes Graves, now in UK; Spiennes, now in Belgium, etc.), and extending significantly in the Bronze Age (e.g., Llandudno in North Wales, UK, and Schwatz and Hallstatt-Dachstein Salzkammergut, now in Austria, etc.) [1,2,3,4]. In Romania, mining activities of ancient peoples were intensified during the time of the Dacian Kingdom (82 BC–106 AD) and even more since the Roman Empire conquest in 106 AD. The Roşia Montană area, within the Arieş River Basin (Romania), includes some of the most famous archaeological sites in the country. This historical site retains various negative effects of those mining operations [5,6,7,8]. During the Middle Ages, the Renaissance, and even the modern era, the mining impact on the environment continued [9,10].

Commonly, mining activities have a negative impact on the environment in general [11,12] and on aquatic ecosystems in particular [13,14,15,16,17]. The effects of this negative impact can be manifested throughout the whole ore extraction and processing cycle [18,19]. The impact can be limited and even ‘beneficial’ when the mining waste and outgoing waters are relatively clean enough (not polluted, or with low contamination), in any case with non-precluding biotic characteristics when compared to the natural conditions. In the case of some abandoned waste (i.e., heaps of waste from mining operations and products processing, or from abandoned settling ponds), a set of very interesting ecosystems can be established or enhanced, consisting of pioneer vegetation, wetlands, forests, thickets, extremophile communities, agricultural land, or recreational spaces [20]. Indeed, some historic mined landscapes (e.g., in Britain and Ireland), exhibit enhanced geographical or biodiversity interest and significance [21,22]. In other cases, waste and waters can be reused for some purposes, but these examples are relatively rare [23].

The negative impact includes spring capture, which can seriously affect the availability and the uses of the waters [24], together with the pollution of the aquifer and running waters, including the leaching of mining waste [25]. Running water pollution can occur at different phases of mining activities, and the effects may persist for decades or even millennia, as is the case of the mining activities in the Arieş River basin [11] caused by the pollutant substances that still remain in situ.

In the mining areas of the Arieș River catchment, where manganese, copper, lead, zinc, cadmium, magnesium, silver, gold, etc., are exploited, the mining industry activities can be divided into four main categories, viz., ore extraction and exploitation; ore processing and mining waste removal; and mine water and post-mining water with uncontrolled discharge of polluted waters. All these categories of mining-related activities overlap or are intermixed, which makes it difficult to identify the main pollutant determining factors. The overlapping effect of these activities also makes it relatively complicated to clearly define a design of the mitigation of negative effects, possible preventative methods and improvement of risk management strategies.

The water quality of the Arieş catchment area, to which the Corna River belongs, is still of permanent concern considering the human impact on the area over thousands of years, especially due to mining activities [10,26], adding to negative effects of other human impact. Although the most modern impacts are still very strong, the new impacts (urban wastewater pollution, fragmentation of the lotic environment due to hydro-technical works, waste deposits on the riverbed or even in the river stream, etc.), are considered to be relatively less important in comparison with the historical local mining effects. The new human pressures combine with the historical ones, generating a high potential environmental impact and inducing significant synergic negative effects on various aquatic communities (aerobic mesophilic heterotrophs, ammonifers, detritivore and iron-reducing bacteria, diatoms, Oligochaeta, Heteroptera, Trichoptera, Turbelaria, Gastropoda, Hirudinea, Crustacea, Coleoptera, Diptera, Ephemeroptera, Megaloptera, Plecoptera, fish, etc.) [27,28,29,30,31,32]. This makes it harder for these organisms to support ecosystem functions, including watershed self-cleaning, and creating a consequent chronic pollution status and significantly facilitating the occurrence of ecological accidents [6].

The optimal integrated management of the resources and services offered by the aquatic ecosystems of the Arieş hydrographical basin, and also the potential risk assessment of the interest area, should be based on studies regarding aquatic and semi-aquatic communities with a role in the lotic ecosystems self-regulation and self-sustainability for every existing sub-basin in correlation with biotope parameters. It is also necessary to assess the cumulative spatial and temporal impact of both pressures (old and new) and threats in the context of different plans for new activities in the reference area, this being the primary objective of the present study regarding the Corna Valley basin lentic and lotic water body area.

The evaluation of the lotic system integrity requires the analysis of the habitat characteristics, together with the vegetation, invertebrate and vertebrate community structures that include key species for the aquatic ecosystems. This evaluation has to be implemented within an approach in a spatial and temporal dynamics context of the population structures and biological dynamic processes characteristic calibrated for the wetland ecosystems [33,34,35,36,37].

For the sustainable management of the resources and services offered by the lotic/lentic systems to the human socio-economic activities it is necessary to assess the functionality and self-regulation capacity of those systems. It is also important to carry out monitoring activities with the prognosis of the related wet/moist/mesic systems trends, in the conditions of past, actual and potential human impact [38,39].

The most important sources of pollution in the Arieş Basin are represented by mining operations in the region Abrud–Câmpeni–Baia de Arieș, metallurgical zones of Turda–Câmpia Turzii area, wood processing installations in the upper basin, the construction materials industry in Turda, pollution with urban waste waters from riparian localities and isolated farms, etc. The mining zone Roșia Montană, Roșia Poieni-Abrud, Baia de Arieș, and Iara is the area with by far the most severe negative impact on the flowing waters of the Arieş Basin, due to long and intensive human impact [40].

Since the Dacian Kingdom, even before the Roman occupation, more than two millennia ago the waters of Arieş Basin were utilized for mining activities (Roşia Montană, Bucium, Baia de Arieş, etc.). The same waters were also used and influenced by forestry logging activities (deforestation, transportation of wood from Apuseni Mountains, water sawmills—e.g., on Iara, Valea Bistrei, and their tributaries)—and by hydraulic milling installations (e.g., on Valea Ierii, Arieşul Mic, and Arieşul Mare). Old uses are indicated by numerous vestiges (ponds in the upper basin of the Roşia Valley show remains and traces of aqueducts and adduction pipelines from the Roman period—e.g., at Lupşa), and by various historical documents [19].

The Mureş River is the largest collector of liquid and solid substances derived from the anthropogenic activities in the Arieş Basin, and is the most exposed to the risks of synergistic impact effects from all the human activities in the area [41].

In particular, the ore mining and preparation industry requires large amounts of water for technological processing. The water is taken from the lotic/lentic systems, and is then directly or indirectly discharged into the Arieş Basin hydrographical network, with greater or lesser alterations of physical, chemical, biological and ecological characteristics [9,42].

The sources of pollution in the area are dictated by the specificity of mining industrial activities: decanted waters from poorly treated ponds; contaminated waters from mining quarries and tailings dumps, which dissolves the active substances found in ores or sterile rocks; mine waters draining from active and former galleries. Water use in technological processes of preparation, flotation, and de-cyanidation bring significant qualitative and quantitative changes to the Arieș River water and some of its tributaries. The most significant impact on the waters of the Arieș River and its tributaries lies with the exploitation of the areas of Roșia Montană, Abrud, and Baia de Arieș [5,9,43].

The Roșia Montană mining area is located in the Abrud River basin, consisting of the right tributary of the Arieș River, in the locality of the same name, at an altitude of ca. 600–700 m, 11 km northeast of the town of Abrud and 15 km southeast of the town of Câmpeni, in Alba County (Transylvania, Romania). The mining objective at Roşia Montană always was the extraction and processing of gold-silver ore. The resulting water input directly into the hydrographic network through tributaries (e.g., Roșia Montană, Săliște, Valea Cornei, Abruzel, Bucium, etc.) in Abrud and later in Arieș and respectively in Mureș Rivers, characterized by high contents of suspensions, fixed residues, CCO_Mn_, sulfates, chlorides and heavy metals, with an acid pH sometimes <3. The largest excessive values are recorded for iron and copper ions, which are hundreds and even thousands of times higher than the legal limits. Significant overshoots are also noted in the suspension, fixed residues, manganese and partly copper and cadmium indicators [43]. Other toxic substances, as for example persistent organic pollutions and pharmaceuticals [44,45,46], impact synergically and diminish the ecological state of the Mureş River, the main tributary of the Tisa River.

Well-known intensive mining areas exist along several tributaries of the Arieş River (Steregoi, Şesei, Muşcanilor, Ştefanca, Roşiei, and Săliştei), and in the Valea Cornei Valley that contains the tailings heap in Cârnic. The infiltration water ponds of Cârnic and the tailings pond, tailings dam and secondary retention system, are also documented [5].

## 2. Materials and Methods

**Figure 1:**
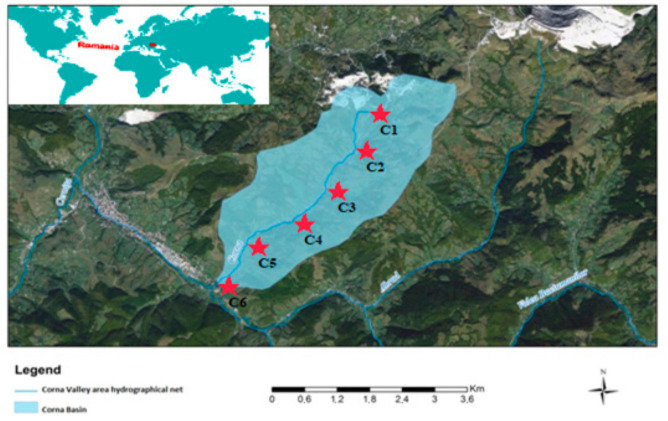
Location, boundaries, and sampling stations (C1
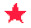
–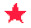
C6) from upstream to downstream of the Corna Valley hydrographic basin.

The Valea Corna valley basin study site (Figure 1) is located in the basin of the Abrud River, an Arieș River tributary.

Ecological methods for identifying the impact of mining activities on aquatic ecosystems are based on the collection and interpretation of samples of benthic macro-invertebrates and fish in correlation with biotope characteristics (hydrological characteristics, the riverbed and banks structures, the aquatic and riparian vegetation type).

For the collection of benthic macroinvertebrates, a self manufactured Surber-type sampler with a surface area of 887 cm^2^ and a 250 μm sieve was used. The collected biological material was fixed in formaldehyde 4% to which NaHCO_3_ was added, and then sorted and identified under a Carl-Zeiss 65x magnifier (Carl Zeiss Microscopy, Oberkochen, Germany). The analyzed material was conserved in 70% alcohol. A self manufactured mountain fishing bag of the Bănărescu type, was used as fish sampler.

To describe the quantitative structure of the Valea Corna benthic macroinvertebrate communities, the relative abundance (A%) and the statistical density (Ds) were used. For the synthetic assessment of the ecological state of the studied lotic sectors the Belgian Biotic Index was used [47].

Five surfaces of 887 cm^2^ were sampled in every sampling station in the spring–summer season; sampling stations were located at 1 km distance from one another to enable highlighting of even the small changes which occur in biotope and biota characteristics from upstream to downstream.

## 3. Results

Analyzing the period 2002 to 2018, several elements regarding the ecological status of the Corna Valley lotic system have been identified. Benthic macroinvertebrate communities are characteristic of the Carpathian rivers of this zone. The plankton cannot grow due to the relatively high water flow speed (if planktonic elements appear, they are of allochthonous origin, coming from some stagnant surrounding/accessory ecosystems—ponds, lakes, marshes, etc.). For the same reasons, the hydro-geomorphological conditions do not allow the development of floating and submerged aquatic macrophytes.

It is important to emphasize that ichthyofauna is absent from the Corna Valley, partly due to the existence of some sectors with small water flows, but especially due the presence of significant historical pollution from mining. Accidentally (once during the study period), two individuals of chub (*Squalius cephalus*), a species resistant to pollution, entered the river a few tens of metres, probably for a short time, but this because of the proximity to the confluence with the Abrudel River.

Table S1 (Annex) shows the structure of the benthic macroinvertebrate communities of the six sectors (C1–C6) of the analyzed Corna Valley, specifying average densities and relative abundances for the individual taxonomic groups.

The analysis of the structure of the benthic macroinvertebrate communities in correlation with some biotope characteristics (hydrological features, the riverbed and bank structures, the aquatic and riparian vegetation type) indicates that the Corna River presents two different sectors of variable ecological status. These sectors and related ecological status are classified as follow: 1. Moderate environmental status on the C1–C5 sector; along this sector, the composition and abundance of benthic invertebrates deviates moderately from the characteristic structure of these communities under natural conditions; 2. Poor ecological status in the sector ca. 1 km upstream of the confluence with Abrudel River (C6); the structure of benthic invertebrate communities is strongly altered compared to the characteristic structure of these communities under natural conditions, the diversity of invertebrates is low, numerically dominant are the Chironomidae larvae, with other groups appearing with relative abundances under 3%.

The structure of benthic macroinvertebrate communities is characteristic of human activities in disturbed places (impacted habitats, landforms, and hydrodynamics modifications) [48,49,50]. The density and specific diversity of the groups Ephemeroptera, Plecoptera and Trichoptera are small if compared to other Carpathian rivers of the same type [44,51,52,53,54].

## 4. Discussion

### 4.1. Pressures

The negative impact in the study area of anthropogenic activities due to the non-ferrous metals (manganese, copper, lead, zinc, cadmium, magnesium, silver, gold, etc.) ore exploitation and processing industry has induced numerous pressures on the environment.

Identification of quality classes for the Corna River that are different to the potential natural state of this river, reveals the presence of various types of significant anthropogenic impact.

The following classes of quality can be distinguished along the Corna River, according to the Belgian Biotic Index [47]: the river sector ranging from C1 to C3 corresponds to the third quality class—polluted waters critical situation; sector C4 corresponds to second class of quality—low polluted water. Improving the ecological status towards the upstream sector may be due to the intake of clean water from tributaries and hydrological conditions (accentuated slope, high flow velocity, and predominant lithological substrate), which favors the processes of natural water self-cleaning of organic matter; sector C5 corresponds to the third class of quality—polluted water critical situation; sector C6 corresponds to the fourth class of quality—waters considered to be highly polluted.

The species of Plecoptera, Ephemeroptera, and Trichoptera identified in the Corna Valley are relatively common species, which are of no conservation interest.

The lack of fish reveals the poor ecological conditions.

**Figure 2:**
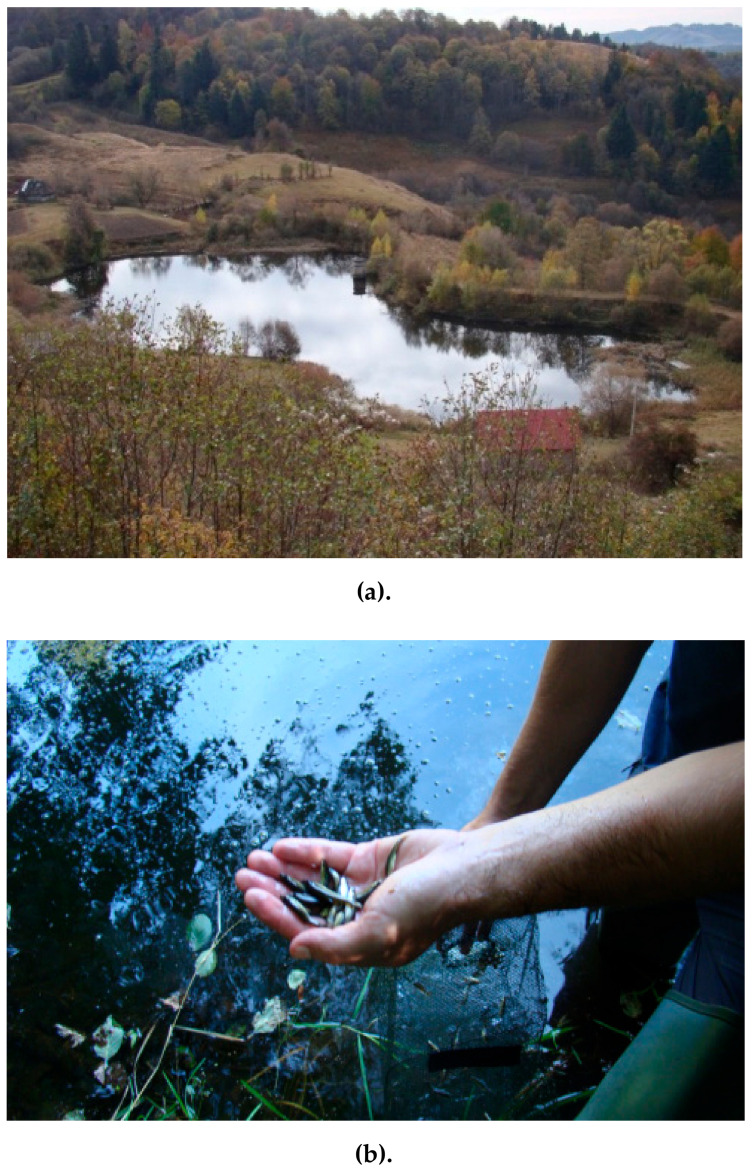

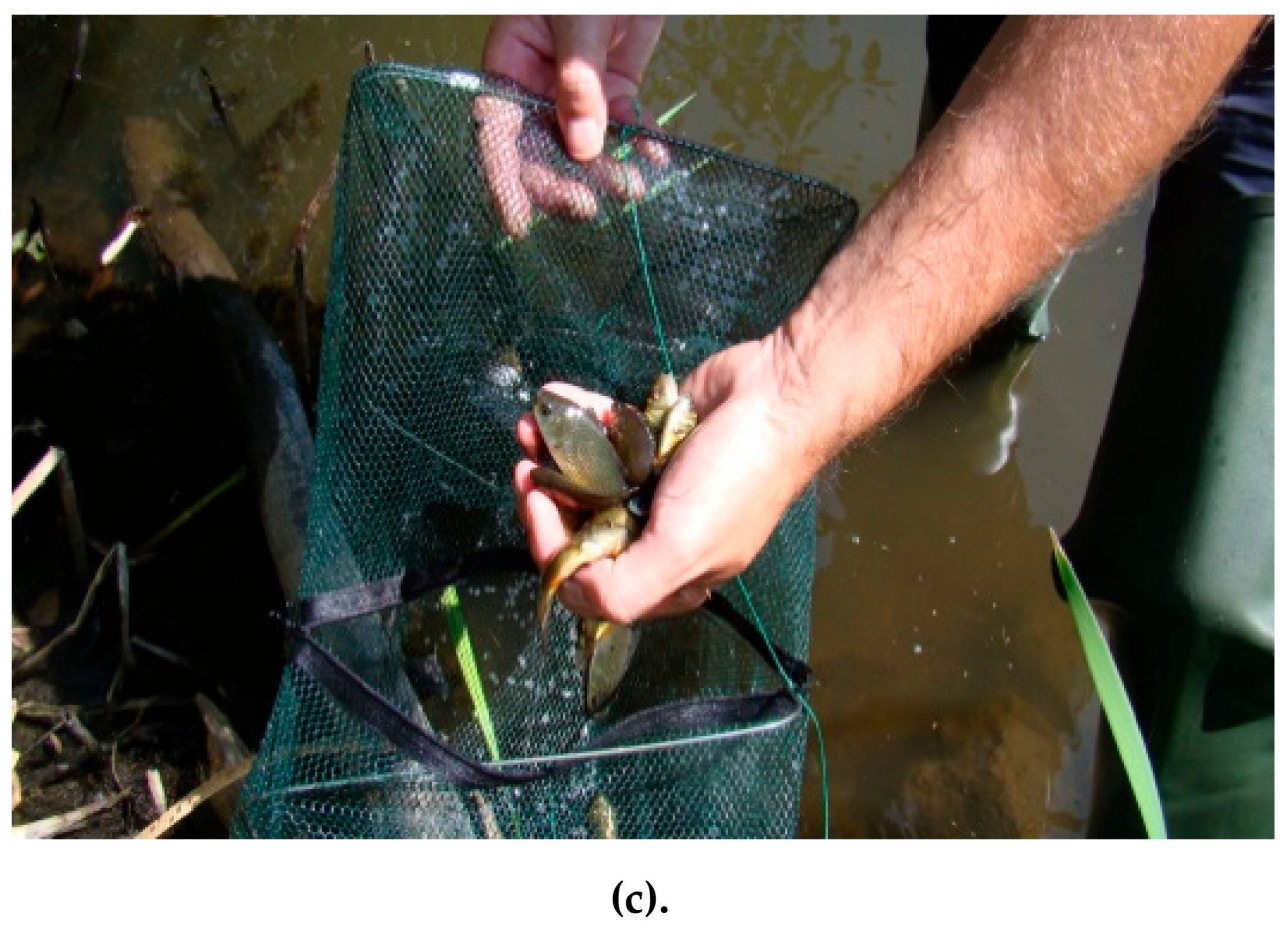
(**a**). Corna Lake, a permanent source of organic matter for the Corna Valley. (**b**). *Pseudorasbora parva* individuals sampled in Corna Lake. (**c**). *Carassius auratus gibelio* individuals sampled in Corna Lake.

Along the entire Corna River there is an intake of organic matter from the upper Corna Lake (Figure 2a) and from the discharge of residual water (urban and zoo-technical) from the houses and farms near the river in the Corna Valley, which do not have adequate sanitation procedures and systems at the moment.

Corna Lake is of anthropogenic origin and was created in the past for the accumulation of groundwater and precipitations, necessary for the processes of grinding and washing ores, in the north-eastern part of the Corna locality, south of Cârnic mining area zone. It has a maximum water depth of 3.6 m, with a bottom covered by a thick layer of clayey mud (thickness > 2 m). This lake is a permanent lentic biotope, which holds an ichthyo-coenosis made up of just two invasive species of no economic and/or conservation importance, topmouth gudgeon (*Pseudorasbora parva* (Temminck and Schlegel, 1846)) (Figure 2b) and Prussian carp (*Carasius auratus gibelio* (Bloch, 1782)) (Figure 2c), which probably arrived as eggs on birds’ feet, and established new populations there.

Past uses of the Corna Lake water for the processing of non-ferrous minerals, actually makes its downstream flowing water relatively high in concentrations of mercury and selenium.

According to physico-chemical analyses of the water, significant pollution is generated mainly by the intake of arsenic, cadmium, chromium, selenium, and sulfate ions, and by the pH (acidic and strongly acidic waters with pH < 3) of some tributaries of the Corna Valley that wash the mining galleries (the confluences are ca. 500 m downstream of Corna Lake and 700 m upstream of the C3 sampling station). The low ecological status of the Corna Valley is mainly due to its origin and tributaries in the zone of old mining areas, from where flows highly acidic water loaded with arsenic, cadmium, total chromium, selenium, and sulfate. Although there are over 70 small springs in this basin, their waters fail to ensure a sufficient dilution. For that reason, in the confluence area between Corna and Abrudel rivers, a number of pollutants remain at a high concentration, especially arsenic, cadmium and selenium. At the same time, the groundwater is highly contaminated with a high ferric hydroxide content, which gives the characteristic yellowish-red color to the water and riverbed.

About the Corna River the presence of a hot geochemical area increases the complexity of elucidating, and therefore possible remedying of the poor ecological status of this area of interest.

Notably the least favorable ecological status for aquatic organisms in the study area is in the lowest sector of the Corna River, a situation which may be influenced in the future by the possible construction of a de-cyanidation dam and lake for mining industry purposes.

It is important to emphasize that constantly low pH values are a well-known factor that induces inhibition of reproduction and growth, and even the death, of intolerant macroinvertebrates.

The variation of macro-invertebrate groups shows a direct link to more or less severe pollution in different sectors of the Corna Valley (Table S1). This type of pollution is generally more pronounced, as the lotic system is smaller and at higher elevation a.s.l., if compared with larger lotic systems located at lower elevation, as confirmed by our research for the Arieș Basin, probably due to differences in abiotic factors (water hardness, alkalinity, temperature, flow, etc.).

Following the actual proposal to try to solve this eco-toxicological problem by barrages and establishment of the de-cyanidation lake, there remains the possibility of physico-chemical release of eco-toxic metals from the initially stable rocks and related minerals. A future pollution wave could produce a negative effect not only as it passes through the Corna lotic system, but also beyond, due to the deposition of the fine sediments with cyanide content carried out by the flow, the inorganic sediments status and regime being a well-documented habitat factor which negatively influences macroinvertebrates and fish [44,55,56].

In the case of an accident (wrong liquid and solid flow management, earthquake, terrorist attack, etc.) at the dam, the consequent pollution wave from the de-cyanidation lake will transport water and sediment along the Corna Valley and into the downstream lotic sectors, impacting not only the existing benthic macroinvertebrates but also their microhabitats, thus affecting negatively their natural and/or artificial re-colonization.

The presence in the sediments of organic matter and sulfides (sphalerite—ZnS, galena—PbS, chalcopyrite—CuFeS_2_, millerite—NiS, arsenopyrite—FeAsS, and cinnabar—HgS) add to the increase in water de-oxygenation and affect the benthic macroinvertebrates, contributing to make this lotic system impoverished in biological communities. The anoxic sediments can immobilize metals in the form of sulfides and thus increasing the toxicity of the aquatic habitats.

Concerning the problem of the suspended sediments, in addition we need to underline how the metals tend to associate with these rather than the water and consequently persist longer in the habitat. The environment created by these toxic sediments is not favorable for aquatic biodiversity.

Excessive and accelerated sedimentation can affect the respiratory organs of Ephemeroptera and Trichoptera, even of those species resistant to pollution and still existing in Corna Valley. In this situation, the low pH values tend to increase the toxicity of the common pollutants: thus, if 4 mg L^−1^ of iron does not have a toxic effect at pH 5.5, an amount of only 0.8 mg L^−1^ of iron at a pH of 4.8 can induce death of fish [57].

The historical presence in the water of suspended solids and sediments, and the bioaccumulation in trophic networks in the long term of some toxic elements, must be one of the most important causes of the low ecological status of the Corna Valley.

The benthic organisms living in environments with contaminated sediments are exposed directly to contaminants and indirectly through contaminated trophic networks. Additional permanent stress levels of invertebrate communities are reinforced by the additional energy consumption required to achieve tolerance in the face of the historic pollution effects, reducing the possibility of adapting to new and sudden stress levels from further accidental extreme pollution events.

For these reasons, the potential presence of cyanide in a pollution wave highlights a major problem for the future ecological status of the aquatic systems downstream of the planned dam, facing the possibility of downstream water and ecosystem contamination events. There are dozens of physical and chemical methods that can remove cyanide (acidification, alkaline chlorination, ion exchange, air cleaning, ozonation, chemical oxidation, natural degradation, evaporation, biodegradation, oxidation with hydrogen peroxide, electro-dialysis, high pressure oxidation, UV photolysis, bacterial oxidation, etc.). Such water de-cyanidation processes should be performed if possible. Consequently, if an accident at the cyanide lake should occur, the water overflow from the lake will contain as low as possible cyanide concentrations.

Accidental air erosion (exceptional storms) or permanent air erosion (due to normal movement of air masses in the area) can dislodge and carry particulate pollutants even at transboundary level, from the lake into water bodies more or less close to it, or into areas from where they will be washed away by precipitations in the related hydrographical net, influencing the environmental status of large areas [58].

**Figure 3:**
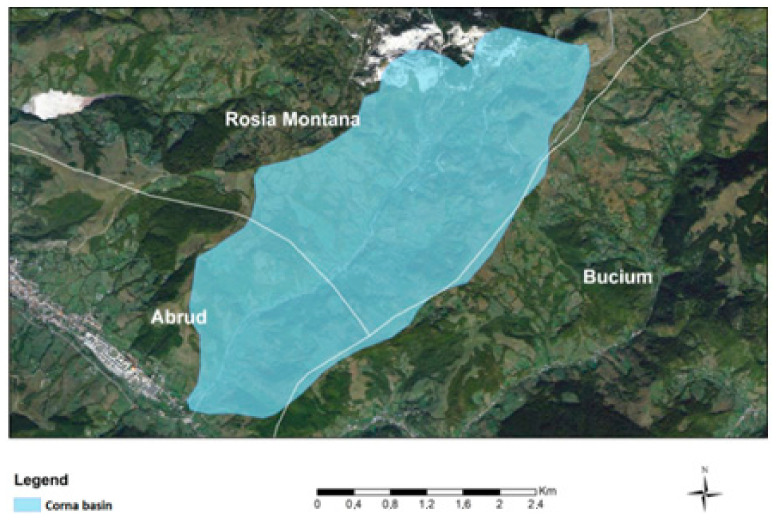
Administrative-territorial units in which the Corna Valley falls: Roşia Montană, Abrud and Bucium.

**Figure 4:**
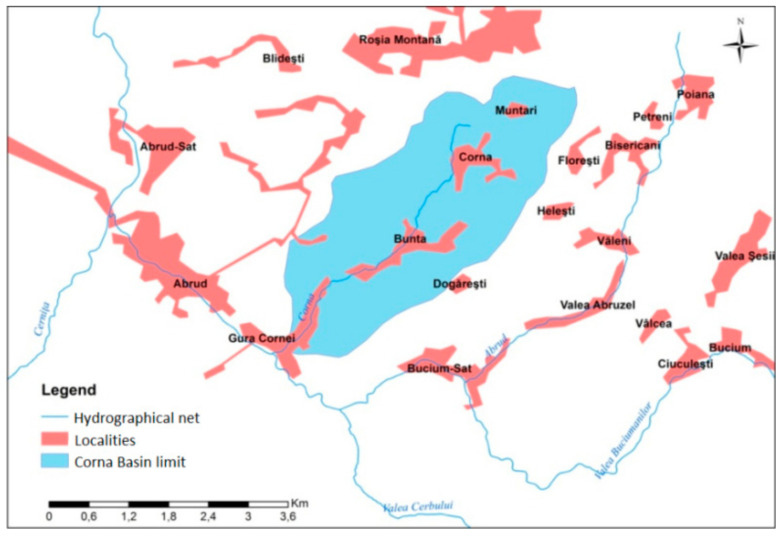
Localities of the Corna Valley and the neighboring areas.

This underlines how the environmental problems identified concern not only the national specific authorities but also the regional and other local authorities on whose territory the Corna Basin is located, the territorial administrative unit Abrud (Figure 3) with the localities Gura Cornei and Abrud (with >5000 inhabitants) (Figure 4). The potential effect of any pollution wave can have the most serious repercussions on the surface water habitats. Repercussions involving groundwater can affect the territorial administrative units of Roșia Montană and Bucium.

**Figure 5:**
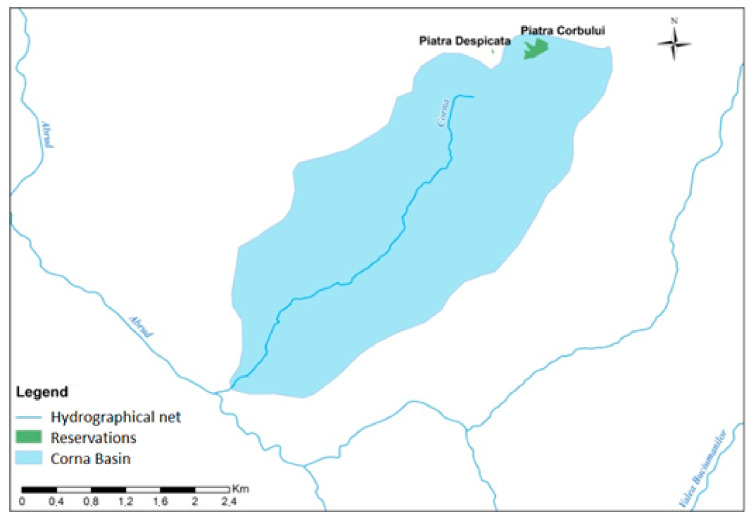
Northern localization of the protected natural elements Piatra Despicată and Piatra Corbului in the context of the Corna Valley basin.

The potential problems identified can have adverse effects only through air influence but not through water movement on the two protected areas of the study area, Piatra Despicată and Piatra Corbului, due to their location upstream of the Corna Valley springs area (Figure 5).

### 4.2. Threats

The following measures are proposed to avoid/minimize/compensate the potential impact of a potential accident with cyanide at the proposed dam of the lake on the Corna Valley.

The water lost from the de-cyanidation reservoir by direct spillage must go through a permanent purification plant with an integrated (automated control and warning) monitoring system, placed immediately beyond the reservoir dam. Thus, the monitoring system could include also the downstream dam areas and the possible barrage infiltration (underground and above, and surroundings), with real-time monitoring and rapid warning capabilities, linked with regional and specialized national institutions. The system must include at least two monitoring stations (one upstream, the second downstream of the reservoir), to carry out comparative analysis of the qualitative status of the liquid flows. Ideally, this monitoring system must also include the ecological assessment stations of the Corna Valley used in the present survey, to provide reference to the initial assessment and a baseline of data prior to the reservoir. The possible combinations of pollutants may trigger mining-specific problems: mine and rock acid drainage, heavy metal contamination, water eutrophication, oxygen depletion, cyanide management issues, etc.

Those factors require permanent monitoring to avoid worse problems. The leaks from the tailing heaps and processed ore heaps may be acid, with a high pollutants content, especially heavy metals; this concern needs to be also a permanent objective for the Corna integrated monitoring system. In the same system must be considered as well the polluting chemical hazards (including cyanide) storage, transfer, and handling areas (for each: identification and monitoring of diffuse sources beside the source points). The ecological status of the aquatic communities, especially of the species/communities considered as indicators, found in the present ecological assessment, must also be included in the proposed integrated monitoring system. In parallel, the structural monitoring (resistance and impermeability) of the dam must be in place and functional. The biological monitoring elements must determine, following specific studies applied to the Corna Valley, whether, due to evolutionary adaptations, the historical exposure of the invertebrate communities to diverse pollution has raised the alert thresholds related to the variation of invertebrate density per unit area in case of an ecological accident. They are presumed to be higher than those of lotic systems not subjected to chronic historical pollution, yet lower due to the situation where the prolonged exposure to chronic pollution cumulatively triggers a weaker resilience of these organisms to an overdose of the same pollutants. Moreover, the biological monitoring must consider, when deciding upon the value of the alert threshold, not only the quantitative issues (number of individuals per surface unit) but also the relation (yet to be identified for this site) between the body size categories of the benthic macroinvertebrates within taxonomic groups and their sensitivity to the increase of the pollution level.

If the costs and/or technical possibilities for building a dam for the formation of a de-cyanidation lake are comparable to those of a negative relief, the latter solution is preferable because the risks associated with accidental damage to the dam disappear. One can take into consideration a mixed option, artificial cavity and rock fill dam (excavated from the artificial cavity), and concrete dam.

The operators of the dam must follow a functional regime and optimal operational parameters and, in case of an accident, to be able to benefit from a real-time alert system of downstream localities, down to the confluence of the Arieş with the Mureş River.

The main measure to avoid/minimize the effects of an environmental accident at the proposed dam of the cyanide lake on Corna Valley would be to create a series of smaller dams to contain accidental cyanide leakage. This solution can be achieved by designing double-rolled downstream pools. These series of dams must be established as close as possible to the main dam, so that the pollution wave is intercepted immediately under the springs and not affect downstream lotic sectors and human communities.

An alternative measure to alleviate accidental leakage from the cyanide lake could be the creation of depressions in the relief (or underground space/trap) with either the ability to store all the polluted water and/or fine sediment resulting from a potential upstream accident, or a smaller capacity and equipped with high-power pumps to remove the liquid and fine sediments back into the lake until the damage has been repaired; other pumps being provided so as to bring clean water out of nearby lakes (their flows to be managed, also during periods of predicted drought) to accelerate the dilution processes of potential overflows to be discharged downstream that cannot be pumped upstream.

These negative relief forms must have a maximum altitude calculated so that other adjacent areas at lower altitudes do not allow the passage of large flows of polluted water, which might flow more into other downstream lotic systems (Abrud, Arieş, etc.). Abandoned mining areas (above or below ground) that exist in the area can also be used for this purpose.

A third proposal could be to decrease the level of the groundwater in order to increase its absorption capacity. This should be done at least for a part of the pollution wave in the proximity of the dam, certainly upstream of the confluence of the Corna Valley with the Abrudel River, in order to avoid the impact of the pollution wave on the downstream aquatic habitats and biocoenoses of the Abrudel, Abrud, and Arieş rivers. The decrease of the groundwater level must be carried out only to the extent that it does not negatively influence the level/volume of the Corna Valley water, either its self-cleaning ability or the permanence of springs and tributaries.

The proposed measurements are desirable because they can be implemented in a complimentary way, reducing secondary risks (for example, a major earthquake might affect both main and secondary dams, in which case only the last two measures would prove effective).

As a measure for slowing the speed of the pollution wave, to prolong the reaction time of the human communities downstream (in the Abrudel, Abrud, and Arieș basins), obstacles for the deviation and/or speed slowdown could be created from natural materials, in the form of large rocks. These should be partially buried to increase their stability and strength on direct impact; placed to fit aesthetically into the landscape; and not sensitive to water containing sulfates.

A compensation measure for the effects of a potential wave of polluted water which can occur (washing and covering the benthic macroinvertebrate communities with polluted sediments), in order to restore the actual Corna Valley’s ecological status, consisting in the trapping of the mobile riverbed minerals, to be sent to a treatment plant (with several washings/treatment of minerals) and re-dispatching them to the place whence they were removed. The very short distance, from the planned dam up to the confluence with Abrudel River, would allow this operation to be carried out within one year. It would be important to work on successive river sectors of up to 100 m, and the work of a new sector should begin only when the next upstream sector has been restored. These activities should be part of the rapid response operational plans at the local and regional integrated levels, carried out at least once every five years by dedicated special teams. They are necessary because, even after the pollution wave has passed, pollutants left in the substrate can subsequently be remobilized in the aquatic environment over time, so following a short, intense pollution event with a series of smaller but longer events.

The measure designed to compensate the negative effects of a pollution wave consists of recolonization with benthic organisms from the Corna Valley, upstream of the de-cyanidation lake, in the area currently in the second category of water quality, after finalizing the implementation of the excavation–treatment–recovery of the river bed mobile minerals, is possible due to the better ecological state of some upstream lotic sectors and the small distances between these sectors.

In order to minimize the impact of solid suspension depositions in the lotic sector downstream of the cyanide lake, it is suggested that sediment traps be constructed, to be periodically emptied and the material deposited in suitable places on the tailings dumps.

Given that suspended sediments containing organic matter and sulfides can contribute to the de-oxygenation of the Corna Valley lotic system, it is proposed that as a measure to minimize their impact, the use of the existing slope gradient by the positioning of local rocky bottom baffles not higher than 20–25 cm from the average water surface, at every 25 m distance one from another.

For the problem of potential excess of sedimentary material in suspension, where metals tend to associate rather than in the water, it is proposed besides the attempt to retain gravitationally these problematic sediments in the tailings lake, increasing the water volume in the Corna Valley downstream of the dam by means of additional flows from nearby basins with cleaner water, which would enhance the self-purification capacity of this lotic system.

To address the issue of the invertebrates still present in the Corna Valley, living in contaminated environments and at additional permanent stress levels from historical pollution, will require a programme for the reconstruction of some of the valley’s habitats. These have improved conditions to provide stress compensation and therefore the possibility of survival of some invertebrates after a pollution accident.

To minimize the effects of erosion and airborne particle transport from the cyanide lake area into nearby water bodies, it is proposed to cover the solidified areas behind the dam with a layer of uncontaminated soil and to assemble and plant grass and shrubs, as well as the creation of dense forestry screens to stabilize and retain as much as possible these polluting particles.

We recommend the integration of all proposed pollution avoidance, minimization and compensation actions and include them in the monitoring and management plans for local and regional watersheds (Arieș and Mureș basins).

## 5. Conclusions

The Corna Valley has one of the oldest historical ecological impacts in Romania due to mining activities.

Fish and benthic macroinvertebrates have proved to have significant response to the ecological effects of long term mining on lotic systems.

From the perspective of the management objectives and measurements required, in the Corna River watershed two management zones can be identified based on the local habitats and biocoenoses ecological assessment: the upper, which can serve as a reference area, and the lower (downstream from the proposed cyanide lake), where direct, comprehensive, and integrated pollution remediation activities should be implemented within a reasonable scale in time and space.

Only a complex set of integrated prevention and remediation methods can avoid a future environmental catastrophic event, as following: on-site adapted designed functional de-cyanidation reservoir, purification plant, monitoring system, and rapid warning systems. Due to the high downstream impact of a possible ecological accident induced by dam damages, supplementary measures of purifying, recirculating, retaining or/and slowing down the pollution wave are essential.

The approach used for this watershed management can be used as a model approach for other similar Carpathian areas, and not only watersheds of conservation interest, with similar significant environmental problems. This type of approach should be based on extensive and intensive biological and ecological data, obtained/monitored at least in a medium-term time period (ten years).

## Data Availability

Any data related to the paper manuscript are available on request; the correspondence author can send the requested data.

## Supplementary Materials

The following are available online at https://www.mdpi.com/1660-4601/18/9/4565/s1, Table S1: Annex.
