# Supplementary material for: Proposed Environmental Risk Management Elements in a Carpathian Valley Basin, within the Roşia Montană European Historical Mining Area
Source: Int J Environ Res Public Health. 2021 Apr 25;18(9):4565. doi: 10.3390/ijerph18094565 (PMC8123413; doi:10.3390/ijerph18094565)
Supplement: Supplementary file 1 [file ijerph-18-04565-s001.zip › ijerph-1176629-supplementary.pdf]

International Journal of
Environmental Research and Public Health

MDPI

Table S1. The structure of benthic macroinvertebrate communities and the description of the river sectors studied along Corna River (Ds – average density, A% – relative abundance).

Taxa	Ds (ind./887 cm²)	A%	The list of the species of the orders Ephemeroptera and Trichoptera
**C1–15 m downstream of Corna Lake**			
In this sector, the maximum width of the minor riverbed is 0.75 m, the minimum width is 0.25 m, the depth of water varies between 20 and 25 cm, the substrate consists of gravel, sometimes there are medium-sized stones; due to the steep slope the water has a relatively high flow speed; on the banks there are trees that create a shadow of 90 – 100% of the minor riverbed. **IBB = 6, the 3rd quality class**			
Taxa	Ds (ind./887 cm²)	A%	The list of the species of the orders Ephemeroptera and Trichoptera
Tricladida	2	0.36	
Oligochaeta	164	30.04	
Gammaridae	323	59.16	
Ephemeroptera	5	0.92	*Baetis vernus* Curtis, 1834<br>*Baetis rhodani* (Pictet, 1843)
Trichoptera	7	1.28	*Glossosoma boltoni* Curtis, 1834<br>*Drusus discolor* (Rambur, 1842)
Diptera<br>Chironomidae	45	8.24	
**C2–1 km aval C1**			
In this sector, the water course is sinuous, the maximum width of the minor bed is 1 m, the minimum width is 0.5 m, the water depth varies between 10 and 20 cm; the substrate is predominantly made up of medium and small sized stones, and boulders, on a mud bed, the coarse sand appears on the banks, on rocks are reddish deposits; low flow rate, rapid areas alternate with lenitic areas; on the banks is arboreal vegetation that creates a shadow of 90 – 100% of the minor river bed. About 500 m upstream of this sector, Corna Valley receives the waters of a stream that washes mining galleries, the water pH is 4,5 – 5. **IBB = 6, 3rd class of quality**			
Taxa	Ds (ind./887 cm²)	A%	The list of the species of the orders Ephemeroptera, Plecoptera and Trichoptera
Oligochaeta	25	20.0	
Gammaridae	11	8.8	
Collembola	3	2.4	
Ephemeroptera	2	1.6	*Baetis vernus* Curtis, 1834<br>*Baetis rhodani* (Pictet, 1843)
Plecoptera	44	35.2	*Nemoura cinerea* (Retzius, 1783)
Trichoptera	3	2.4	*Glossosoma boltoni* Curtis, 1834<br>*Drusus discolor* (Rambur, 1842)<br>*Sericostoma flavicorne* Schneider, 1845
Coleoptera	2	1.6	
Diptera<br>Chironomidae<br>Other dipterans	 31<br>4	 24.8<br>3.2	
**C3–1 km aval C2**			
In this sector, the width of the minor bed of Corna Valley varies between 0.5 and 2.5 m, the depth of water varies between 15 and 25 cm; the substrate consists of fine and coarse sand, gravel, sometimes there are large and medium stones; on the banks is alder gallery, which generates 100% shadow. Approximately 700 m upstream, Corna Valley receives the waters of a stream that washes the mining galleries, with pH 2.5. **IBB = 6–3rd quality class**			
Taxa	Ds (ind./887 cm²)	A%	The list of the species of the orders Ephemeroptera, Plecoptera, and Trichoptera
Oligochaeta	7	18.42	
Copepoda	5	13.16	
Gammaridae	1	2.63	
Ephemeroptera	7	18.42	*Baetis vernus* Curtis, 1834<br>*Baetis rhodani* (Pictet, 1843)<br>*Seratella ignita* (Poda, 1761)
Trichoptera	3	7.89	*Glossosoma boltoni* Curtis, 1834<br>*Plectrocnemia conspersa* Curtis, 1834<br>*Sericostoma flavicorne* Schneider, 1845<br>*Berea pullata* Curtis, 1834
Diptera<br>Chironomidae<br>Other dipterans	 14<br>1	 36.84<br>2.63	
**C4–1 km aval C3**			

Taxa	Ds (ind./887 cm²)	A%	The list of the species of the orders Ephemeroptera, Plecoptera and Trichoptera
In this sector, the average width of the Corna Valley is 2 m, (maximum 3.5 m and minimum 2 m), the average depth of the river is 20 cm (maximum 40 cm and minimum 15 cm); the substrate of the riverbed is made up of boulders, medium and small stones on a mud bed; on the banks of the river there is willow and alder gallery, this vegetal corridor giving 90 – 100% shadow. **IBB = 7 – 2nd class of quality**			
Taxa	Ds (ind./887 cm^2)	A%	The list of the species of the orders Ephemeroptera, Plecoptera and Trichoptera
Tricladida	4	1.01	
Oligochaeta	5	1.26	
Gammaridae	29	7.30	
Ephemeroptera	22	5.54	*Baetis rhodani* (Pictet, 1843)<br>*Ecdyonurus dispar* (Curtis, 1834)<br>*Rhithrogena semicolorata* (Curtis, 1834)
Plecoptera	5	1.26	*Nemoura cinerea* (Retzius, 1783)<br>*Perla marginata* (Panzer, 1799)
Trichoptera	5	1.26	*Glossosoma boltoni* (Curtis, 1834)<br>*Plectrocnemia conspersa* (Curtis, 1834)<br>*Sericostoma flavicorne* (Schneider, 1845)<br>*Hydropsyche pellucidula* (Curtis, 1834)
Diptera<br>Chironomidae<br>Other dipterans	 322<br>5	 81.11<br>1.26	
**C5–1 km aval C4**			
In this sector of Corna Valley, the average width of the minor riverbed is four m (maximum five m), the average water depth of the river is 15 cm (maximum 25 cm); the riverbed is made up of medium and small stones on mud; on the banks is an alder and willow gallery, interrupted in the vicinity of the houses and farms. **IBB = 6 – 3rd class of quality.**			
Taxa	Ds (ind./887 cm^2)	A%	The list of the species of the orders Ephemeroptera, Plecoptera and Trichoptera
Tricladida	1	0.21	
Oligochaeta	82	17.56	
Gammaridae	81	17.34	
Ephemeroptera	96	20.56	*Baetis rhodani* (Pictet, 1843)<br>*Baetis vernus* (Curtis, 1834)
Plecoptera	5	1.07	*Perla marginata* (Panzer, 1799)
Trichoptera	14	3.00	*Glossosoma boltoni* (Curtis, 1834)<br>*Plectrocnemia conspersa* (Curtis, 1834)<br>*Hydropsyche pellucidula* (Curtis, 1834)
Diptera<br>Chironomidae<br>Other dipterans	 184<br>4	 39.40<br>0.86	
**C6–at the confluence with Abrudel River**			
In this sector, the average width of the minor riverbed is one m (maximum 1.25 m), the average water depth is 15 cm (maximum 25 cm, minimum five cm); the riverbed is formed of boulders and gravel, small sandy surfaces appear near the banks; on the banks is a willow and alder gallery, which generates 90 – 100% shadow. **IBB = 4 – 4th class of quality**			
Taxa	Ds (ind./887 cm^2)	A%	The list of the species of the orders Ephemeroptera and Plecoptera
Oligochaeta	3	0.28	
Gammaridae	7	0.65	
Ephemeroptera	32	2.99	*Baetis rhodani* (Pictet, 1843)<br>*Seratella ignita* (Poda, 1761)
Diptera<br>Chironomidae<br>Other dipterans	 1015<br>15	 94.68<br>1.40	
